# Establishing a reference range for thromboelastography maximum amplitude in patients administrating with antiplatelet drugs

**DOI:** 10.1002/jcla.23144

**Published:** 2019-12-06

**Authors:** Wei Shen, Jing‐Yi Zhou, Yi Gu, Wen‐Yan Shen, Min Li

**Affiliations:** ^1^ Department of Laboratory Medicine Renji Hospital Shanghai Jiaotong University School of Medicine Shanghai China

**Keywords:** antiplatelet agents, aspirin, clopidogrel, thromboelastography

## Abstract

**Objective:**

We aimed to establish the reference range of thromboelastograph (TEG) maximum amplitude (MA) in patients taking antiplatelet drugs.

**Methods:**

Between August 2015 and July 2018, a total of 4614 patients administrating with antiplatelet drugs (clopidogrel and aspirin) were retrospectively analyzed in this study. For MA_A_ parameter, we used the 10th and 90th percentiles to establish a reference range. The Spearman correlation was used for the correlation analysis among the inhibition rate of adenosine diphosphate (ADP%) and MA_ADP_, inhibition rate of arachidonic acid (AA%) and MA_AA_. Then, through receiver operating characteristic (ROC) curve analysis of the best cutoff point, the reference ranges of MA_ADP_ and MA_AA_ could be deduced. Consistency evaluation was performed by statistical analysis of ADP% and MA_ADP_, AA% and MA_AA_ pairing for 4459 patients.

**Results:**

The reference range of MA_A_ was 8.1‐25.8 mm. The reference range of MA_ADP_ was 19.8‐43.2 mm, and the corresponding sensitivity of two endpoints was 0.796, 0.856 and specificity were 0.897, 0.904, respectively. The reference range of MA_AA_ was 18.9‐37.7 mm, and the corresponding sensitivity of two endpoints was 0.819, 0.829 and specificity were 0.922, 0.896, respectively. The inconsistency rate of ADP% and MA_ADP_, and AA% and MA_AA_ was 20.1% (898 cases) and 16.6% (738 cases), respectively.

**Conclusions:**

The reference range of MA_ADP_ and MA_AA_ established by us were better in sensitivity and specificity. MA_ADP_ and MA_AA_ were more accurate than conventional inhibition rate analysis in guidance of antiplatelet therapy, especially in patients with excessive low MA or high MA_A_.

## 1 INTRODUCTION

Thrombus is one of the most common lethal disease in non‐malignant diseases, including myocardial infarction, cerebral infarction, pulmonary embolism, and so on.^1^ Once onset, long‐term anticoagulant therapy is needed.^2^ For arterial thrombus, long‐term treatment with antiplatelet agents is usually required, such as aspirin and clopidogrel.^3^ It is necessary to monitor the blood coagulation functions of patients administrating with antiplatelet drugs to test the effectiveness of the drug.

Routine coagulation tests (such as activated partial thromboplastin time (APTT), prothrombin time (PT), and platelet count) are the most commonly used method for evaluating coagulation function. These tests are often used as a starting place when investigating the cause of bleeding. However, routine tests possess only limited capacities to reveal patient's risk of bleeding and do not provide information on the risk for thrombus.^4^ Besides, they do not provide specific data about clot quality or stability.^5^, ^6^

In comparison to the conventional tests, the thromboelastograph (TEG) hemostasis analyzer system can objectively reflect the blood clotting, fibrinogen/fibrin/platelet interactions, and the processes of formation, development, and dissolution of thrombus in the body.^7^, ^8^, ^9^, ^10^, ^11^ TEG can reflect the function of platelet.^12^, ^13^ The therapeutic effect of antiplatelet drugs can be evaluated by adding ADP or AA inducer and deducting the coagulation effect of fibrin reticulum. Maximum amplitude (MA) parameters reflect the strength of blood clots.^14^ The strength of the blood clot is composed of platelet aggregation, contraction, and fibrin network.^15^ Platelets account for about 80%, and fibrin network accounts for about 20%. According to different inducers and detection types, MA can be divided into four types, including Kaolin activity (MA_CK_), fibrin activity (MA_A_), ADP‐stimulated platelet activity (MA_ADP_), and AA‐stimulated platelet activity (MA_AA_).^16^, ^17^

It is generally believed that drugs are more effective when platelet function is inhibited by more than 50%.^18^ According to clinical experience and manufacturer's recommendation, our hospital has established a reference range of 40%‐90% induction inhibition rate of ADP and 50%‐90% induction inhibition rate of AA. However, the platelet inhibition rate needs to consider the MA_CK_ value of the common detection and the MA_A_ value of the fibrin network. When these two values are too high or too low, the calculated inhibition rate will be affected. Therefore, this study retrospectively analyzed the previous thromboelastograph (TEG) data, aiming to directly establish a reference range of MA_A_, MA_ADP_, and MA_AA_, and guide the rational use of drug in clinical practice.

## 2 METHODS

### 2.1 Patients

We retrospectively reviewed 4614 patients administrating with antiplatelet drugs (clopidogrel and aspirin), who were treated in Renji Hospital Affiliated to Medical College of Shanghai Jiaotong University from August 2015 to July 2018. The inclusion criteria were as follows: (a) patients administrating with both aspirin and clopidogrel; (b) patients undergoing the TEG test; and (c) patients having complete TEG data. Considering the abnormal activation of platelet (MAA > 35 mm), the original data with MA_A_ >35 mm were excluded. Finally, the remaining 4459 cases were included in the subsequent analysis. This study was approved by the ethics committee of Renji Hospital Affiliated to Medical College of Shanghai Jiaotong University. Written informed consent was obtained from each patient.

### 2.2 TEG analysis

Platelet‐fibrin clot strength measurements were carried out using the TEG Hemostasis System (Haemoscope Corporation). The TEG Hemostasis Analyzer with automated analytical software provides quantitative and qualitative measurements of the physical properties of a clot.

All analyses were performed with TEG disposable cups and pins as devised by the manufacturer and measurements were performed within 4 minutes of sampling. Briefly, a stationary pin is suspended into an oscillating cup that contains the whole blood sample. As the blood clots, it links the pin to the cup. Clot strength is determined by measuring the amplitude of the rotation of the pin, which increases proportionally with clot strength. Maximum amplitude represents maximum clot strength, expressed as the MA parameter.

### 2.3 Statistical analysis

All statistical analyses were performed by using SPSS version 17.0 (SPSS Institute). The normality of distribution of continuous variables was tested by one‐sample Kolmogorov‐Smirnov test. Continuous variables with normal distribution were presented as means ± standard deviation (SD); non‐normal variables were reported as median (interquartile range [IQR]). For MA_A_ parameter, we used the 10th and 90th percentiles (P_10_‐P_90_) to establish a reference range. The Spearman correlation was used for the correlation analysis among the ADP% and MA_ADP_, AA% and MA_AA_. Then, through receiver operating characteristic (ROC) curve analysis of the best cutoff point, the reference ranges of MA_ADP_ and MA_AA_ could be deduced. Consistency evaluation was performed by statistical analysis of ADP% and MA_ADP_, AA% and MA_AA_ pairing for 4459 patients. Statistical significance was set at *P* < .05.

## 3 RESULTS

### 3.1 Demographic characteristics

We retrospectively reviewed 4614 patients administrating with clopidogrel and aspirin, who were treated in our department from August 2015 to July 2018. Considering the abnormal activation of platelet (MAA > 35 mm), the original data with MA_A _>35 mm were excluded. Finally, the remaining 4459 patients (2512 males, 1947 females; mean age: 54.4 years; range from 39 to 85 years) were included in the subsequent analysis. The baseline characteristics of included patients are showed in Table 1.

**Table 1:** Baseline characteristics of included patients

Characteristic	Number (n, %)
Mean age (range)	54.4 (39‐85)
Sex	
Male	2512 (56.3)
Female	1947 (43.7)
Department	
Department of Cardiology	2756 (61.8)
Neurosurgery Department	250 (5.6)
Neurology Department	699 (15.7)
Vascular Surgery Department	540 (12.1)
Others	214 (4.8)

### 3.2 Establishment and evaluation of MA_A_ reference range

**Figure 1:**
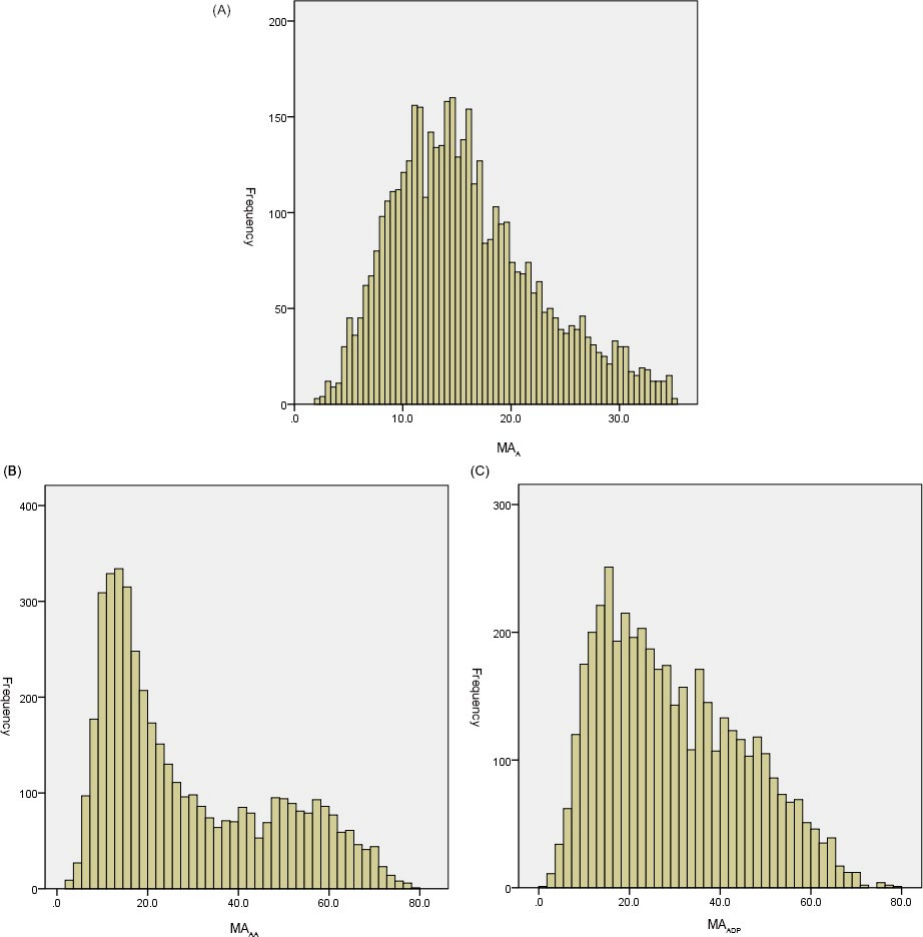
Data distribution of MA_A_, MA_AA_, and MA_ADP_. ADP‐stimulated platelet activity; MA_A_, fibrin activity; MA_ADP_, MA_AA_, AA‐stimulated platelet activity

Considering the skewed distribution of MA_A_ values (Figure 1A), the reference range was 8.1‐25.8 mm (P_10_‐P_90_). To further evaluate the feasibility of the MA reference range, TEG results of 155 patients (MA_A_ ≥ 30 mm) were retrospectively analyzed. Only 11 cases did not show large opening pattern. Especially for patients with MA_A _≥60 mm, the inhibition rate showed a higher false value (Table 2).

**Table 2:** Analysis of the inhibition rate of five patients with MA_A_ ≥ 60 mm before and after correction

Case	MA_CK_ (mm)	MA_A_ (mm)	MA_ADP_ (mm)	MA_AA_ (mm)	ADP%		AA%	
					Original	Corrected^a^	Original	Corrected^a^
1	75.0	66.6	69.4	70.2	66.7	9.3	57.1	8.0
2	72.7	60.0	69.0	64.1	29.1	6.4	67.7	14.9
3	77.0	65.9	61.1	32.4	100.0	25.6	100.0	71.8
4	70.6	60.5	60.8	61.2	97.0	17.6	93.1	16.9
5	72.3	60.4	51.1	61.7	100.0	36.9	89.1	18.5

Abbreviations: AA, arachidonic acid; ADP, adenosine diphosphate; MA, maximum amplitude; MA_CK_, Kaolin activity; MA_A_, fibrin activity; MA_ADP_, ADP‐stimulated platelet activity; MA_AA_, AA‐stimulated platelet activity.

a Corrected data: unified calculation with MA_A_ = 14.9 mm.

### 3.3 Establishment of MA_ADP_ and MA_AA_ reference range

**Figure 2:**
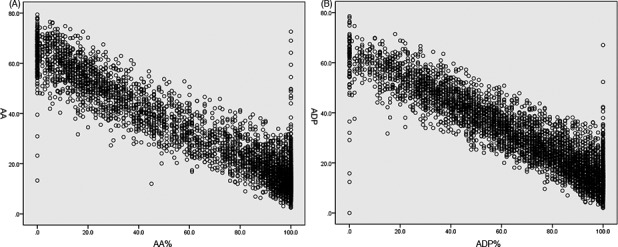
Correlation analysis. A, Correlation analysis between MA_AA_ and AA%. AA, arachidonic acid; MA_AA_, AA‐stimulated platelet activity. B, Correlation analysis between MA_ADP_ and ADP%. ADP, adenosine diphosphate; MA_ADP_, ADP‐stimulated platelet activity

The values of MA_AA_ and MA_ADP_ were skewed distributed (Figure 1B,C). To establish the reference range of MA_ADP_ and MA_AA_, the Spearman correlation was used for the correlation analysis among the parameters firstly. MA_AA_ was significantly negative correlated with AA% (*r* = −.864, *P* < .001) (Figure 2A). Besides, a significant negative correlation was also found between MA_ADP_ and ADP% (*r* = −.892, *P* < .001) (Figure 2B).

**Figure 3:**
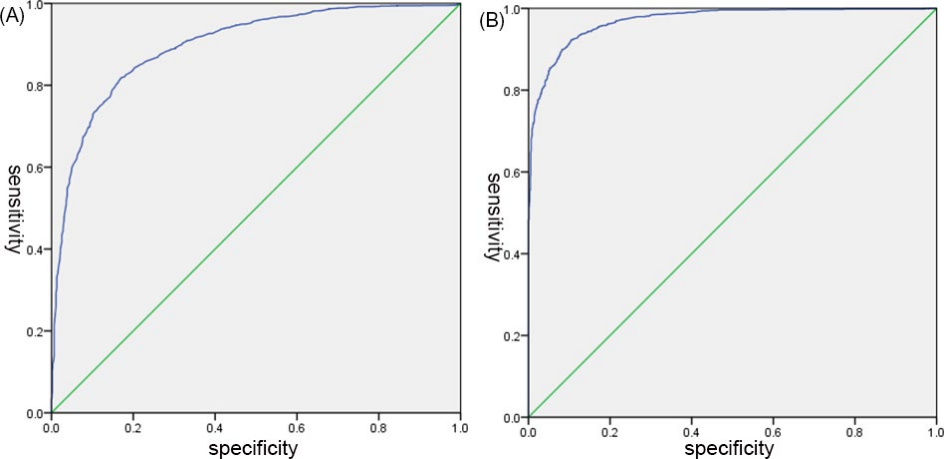
ROC curve of AA%. A, ROC curve of 3086 patients with AA% ≥ 50%; B, ROC curve of 2284 patients with AA% ≤ 90%. AA, arachidonic acid; ROC, receiver operating characteristic

For MA_AA_, ROC curves (Figure 3) of the dichotomous data MA_AA_ and AA% (with [50, 90] as normal) were analyzed and the best cutoff points were found. The ROC curve analysis results showed that AA% was a predictor of MA_AA_ (AA%≥50: AUC = 0.896; sensitivity, 0.819; specificity, 0.922. AA% ≤90: AUC = 0.971; sensitivity, 0.829; specificity, 0.896). The best cutoff values of AA% for MA_AA_ were 18.9 (AA% ≥50) and 37.7 (AA% ≤90), respectively. The reference range of MA_AA_ was 18.9‐37.7 mm.

**Figure 4:**
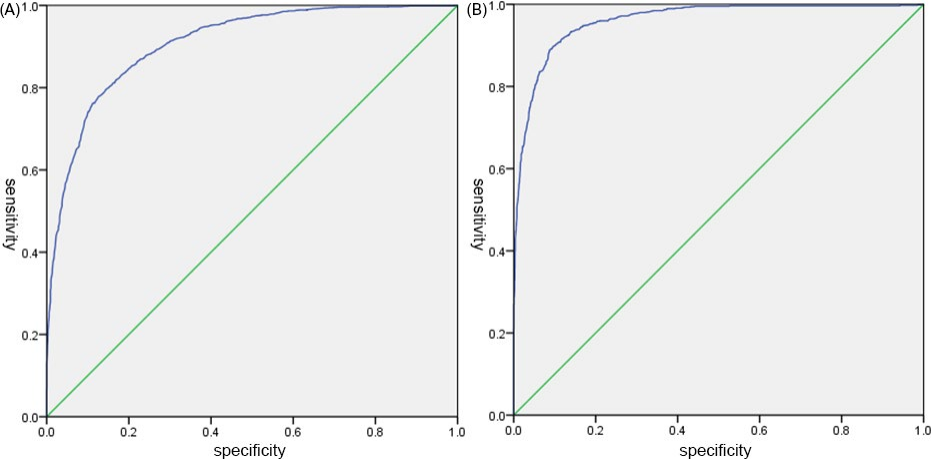
ROC curve of ADP%. A, ROC curve of 3612 patients with ADP% ≥ 40%; B, ROC curve of 3035 patients with ADP% ≤ 90%. ADP, adenosine diphosphate; ROC, receiver operating characteristic

For MA_ADP_, ROC curves (Figure 4) of the dichotomous data MA_ADP_ and ADP% (with [40, 90] as normal) were analyzed and the best cutoff points were found. The ROC curve analysis results showed that ADP% was a predictor of MA_ADP_ (ADP% ≥40: AUC = 0.989; sensitivity, 0.796; specificity, 0.897. ADP% ≤90: AUC = 0.960; sensitivity, 0.856; specificity, 0.904). The best cutoff values of ADP% for MA_ADP_ were 19.8 (ADP% ≥40) and 43.2 (ADP% ≤90), respectively. The reference range of MA_ADP_ was 19.8‐43.2 mm.

### 3.4 Feasibility analysis of MA_ADP_ and MA_AA_ reference range

To further evaluate the feasibility of the MA reference range, consistency evaluation was performed by statistical analysis of ADP% and MA_ADP_, AA% and MA_AA_ pairing for 4459 patients. The results showed that the inconsistency rate of ADP% and MA_ADP_ was 20.1% (898 cases), AA% and MA_AA_ was 16.6% (738 cases). Through the analysis of inconsistent cases, it was found that there was a significant correlation with MA_A_ and MA_CK_ values. When the MA_A_ and MA_CK_ values were too large or too small, the inconsistency rate increased significantly (Table 3). Such as, for the smallest 100 cases of MA_A_, the inconsistency rate of ADP% and MA_ADP_ was 30%, AA% and MA_AA_ was 37%, which was significantly higher than the median 100 cases of MA_A_ (the inconsistency rate of ADP% and MA_ADP_ was 3%, AA% and MA_AA_ was 2%).

**Table 3:** Comparisons of the inconsistency rate between MA_ADP_ and ADP%, MA_AA_ and AA%

Cases (n = 100)	MA Value Interval (mm)	IR between MA_ADP_ and ADP%	IR between MA_AA_ and AA%	Explanation
MA_A_ (minimum)	2.1‐5.2	30.3	37.1	Inhibition rates were in the appropriate range, MA_ADP_ or MA_AA_ was below the reference range
MA_A_ (median)	14.8‐15.1	2.9	2.1	‐
MA_A_ (maximum)	31.6‐35.0	40.0	36.2	MA_ADP_ or MA_AA_ was in the reference range or slightly beyond the upper limit, and the inhibition rate was beyond the appropriate range
MA_CK_ (minimum)	12.9‐45.9	19.9	29.0	MA_ADP_ or MA_AA_ was in the reference range, inhibition rate was below the appropriate range
MA_CK_ (median)	63.1‐63.5	2.1	2.0	‐
MA_CK_ (maximum)	76.1‐81.7	14.2	10.3	Inhibition rates were in the appropriate range, MA_ADP_ or MA_AA_ was beyond the reference range

Abbreviations: AA, arachidonic acid; ADP, adenosine diphosphate; IR, inconsistency rate; MA, maximum amplitude; MA_CK_, Kaolin activity; MA_A_, fibrin activity; MA_ADP_, ADP‐stimulated platelet activity; MA_AA_, AA‐stimulated platelet activity.

## 4 DISCUSSION

In recent years, the TEG has attracted much attention in assessing blood coagulation function and guiding blood transfusion in the preoperative period. Compared with routine coagulation function tests, TEG can reflect the first and second stages of hemostasis and fibrinolysis and can also reflect clinical bleeding more sensitively.^19^, ^20^, ^21^ The TEG can be more comprehensive and effective in evaluating the coagulation status. According to TEG manufacturer's instructions, the acquisition of ADP% and AA% depended on MA_A_ and MA_CK_ values. However, in practice, when the two values were abnormal, the accuracy of inhibition rate would be affected. For example, when MA_A_ was abnormally increased, the inhibition rate would be falsely increased (Table 1). Clinical workers also explored the direct evaluation of MA_ADP_ or MA_AA_ to guide clinical application. For example, the study of Sinai Hospital in Baltimore, USA, tracked 225 patients who received percutaneous coronary intervention (PCI). It was considered that MA_ADP_ >47 mm was a predictor of re‐infarction risk, while MA_ADP _<31 mm was prone to bleeding risk.^22^ However, the risk value was not equivalent to the reference value of whether the drug was effective. So our laboratory directly evaluated the MA_ADP_ or MA_AA_ reference range of whether the drug was effective by retrospective analysis of previous results of 4614 patients administrating with antiplatelet drugs (clopidogrel and aspirin). The results showed that the reference range of MA_ADP_ and MA_AA_ established by us were better in sensitivity and specificity. MA_ADP_ and MA_AA_ were more accurate than conventional inhibition rate analysis in guidance of antiplatelet therapy, especially in patients with excessive low MA or high MA_A_. Besides, a significant negative correlation was also found between MA_ADP_ and ADP%.

Since the reference range was negatively correlated with the inhibition rate, attention should be paid to clinical use. Exceeding the upper limit of the reference range indicated poor drug efficacy, and the closer the result was to the MA_CK_ value, the worse the efficacy was. Below the limit of the reference range, the original inhibition rate exceeded 90%, indicating that the drug was very effective, but did not indicate whether there was a risk of bleeding. Especially in the clinical use of antiplatelet membrane glycoprotein IIb/IIIa classes of antiplatelet aggregation drugs,^23^ the fibrin and platelet junction sites were inhibited. The MA_ADP_ values and MA_A_ values were almost identical, and below the limit of the reference range only showed that the drug was effective, but did not indicate whether there was a risk of bleeding.

By analyzing the cases of ADP% and AA% inconsistent with MA_ADP_ and MA_AA_, we found that when the values of MA_A_ and MA_CK_ were too large or too small, the inconsistency rate increased significantly (Table 2). For the smallest 100 cases of MA_A_, it was found that the inhibition rate was generally calculated in the appropriate range, MA_ADP_ or MA_AA_ was below the reference range, and the ADP and AA inconsistency rates were 30% and 37%, respectively. However, they all suggested that the drug was effective and therefore did not affect clinical treatment. However, for the largest 100 cases of MA_A_, it was found that if the MA_A_ was falsely increased, the inhibition rate would incorrectly indicate that the drug was effective.

Since the MA_A_ value was easy to affect the calculation of inhibition rate, it was necessary to establish a reference range. Only when the MA_A_ value was in a reasonable range, the calculation of inhibition rate would be more reliable. MA_A_ reflected the patient's fibrin formation ability. When the value was too high and the TEG tracing continues to grow wider, it reflected the abnormal activation of platelets. It was recommended to try glycoprotein IIb/IIIa inhibitor.^24^

This study has several limitations. Firstly, this study was a retrospective, single‐center and non‐control study, which had certain limitations in clinical application. Secondly, this study only studied the MA parameters and did not analyze other TEG parameters such as r‐time, k‐time, and α‐angle.^25^ Therefore, multi‐centered prospective studies should be conducted in the future to establish a more accurate reference range of TEG in patients taking antiplatelet drugs.

In conclusion, TEG could be used as a relatively reliable method for monitoring antiplatelet drugs. Compared with the traditional inhibition rate analysis, the direct establishment of MA_A_, MA_ADP_, and MA_AA_ reference range could be more rationally in guide the use of drug in clinical practice.
